# Reduced predator avoidance follows ocular and lateral line pathology in minnow species after diesel exposure

**DOI:** 10.1038/s41598-026-53051-z

**Published:** 2026-05-26

**Authors:** Matthew Bolerjack, Paula A Schaffer, Pete Cadmus

**Affiliations:** 1 Colorado Parks and Wildlife, Aquatic Research Section, Fort Collins, CO 80526 USA; 2 College of Veterinary Medicine and Biological Sciences, Department of Microbiology, Immunology and Pathology, Colorado State University, Fort Collins, CO 80523 USA

**Keywords:** Diesel, Keratitis, Uveitis, Lateral line, Predator avoidance, Ecology, Ecology, Environmental sciences, Zoology

## Abstract

Petroleum chemicals reach freshwater habitats and can elicit acute toxic effects on ecosystem members.^1^Until recently standardized acute toxicology experiments and water quality standards (also referred to as Maximum Allowable Concentrations in some jurisdictions) focused on mortality of aquatic organisms after 96 h of toxicant exposure^2,3^, but in nature sublethal effects can manifest into mortality and in lotic environments (streams and rivers) exposure times can be far shorter (< 24 h). Here we show that Plains Minnow (*Hybognathus placitus*), Fathead Minnow (*Pimephales promelas*), and Flathead Chub (*Platygobio gracilis*) (Family Leuciscidae) exposed to sublethal concentrations of diesel fuel for 24 h experienced sublethal injuries which render them susceptible to death by predation. Diesel-exposed prey represented 77% of predated fish across all predation events. Decreased predator avoidance likely arose from sensory impairment. Diesel-exposed Fathead Minnow had significantly decreased lateral line neuromast counts and severe ophthalmic pathology. Standardized toxicity experiments would have missed these key effects. Sublethal petroleum exposures have global implications for the conservation of fish species and derivations of water quality standards intended to protect fish from petrochemical pollution need to include sublethal endpoints with the potential to manifest into population loss.

**Supplementary Information:**

The online version contains supplementary material available at 10.1038/s41598-026-53051-z.

## Introduction

Many forms of diesel fuel are used globally in all forms of transportation and the constituents of diesel are common in other fuels, petrochemicals, and crude oils. Petroleum spills are frequent anthropogenic disturbances across the world^1^. Fuels (petrol/gasoline, and diesel oils) from highway accidents and spills from well sites and pipelines enter freshwater ecosystems by accident, while produced water discharge from oil and gas wells and urban stormwater runoff with fuel contaminants drain to freshwater ecosystems by design^4^. Policy has helped decrease some spill events, but spills remain a persistent problem due to growing population and energy demands.

Crude oil and refined products like diesel fuel are mixtures of numerous classes of chemicals including polycyclic aromatic hydrocarbons (PAHs), benzene, naphthenes (cycloalkanes), and metals^5,6^. Each chemical component has a different fate (retention and loss) and bioavailability in aquatic ecosystems or experimental aquaculture tanks. During spill events fuel mixtures are often simplified to total petroleum hydrocarbons (TPH, mg/L) of diesel range organics (DRO) based on carbon chain lengths (C10–C28), while laboratory experiments used to prescribe environmental risk are typically conducted using a single petrochemical component at a time. Single component studies offer more control and repeatability but fail to evaluate possible synergistic effects of fuel components.

Jurisdictions generally regulate pollution to stay below a threshold (water quality standard or criterion) that protects biodiversity in “acute” short-term exposure regimes (e.g., Criterion Maximum Concentration, Maximum Allowable Concentration) or “chronic” long-term persistent exposure regimes (e.g., Criterion Continuous Concentration, Annual Average). Historically, the experiments that informed chronic studies considered mortality as well as sublethal response variables that could reduce fitness of test organisms. In contrast, standardized acute toxicity experiments used to inform risk assessment and water quality standards have almost exclusively considered the mortality of animals after 96 h of toxicant exposure^2,3^. However, sublethal exposures to toxicants can impair physiology and behaviors needed for reproduction, social interactions, predator–prey dynamics, and other functions and thus can impair fitness^7–10^. Sublethal impairments have potential to result in mortality or population loss in nature after a short term exposure event. For this reason, more modern regulatory systems consider sublethal endpoints in derivation of pollution limits. We explored reduced predator avoidance, a potentially more sensitive sublethal toxic response that may result in mortality after an exposure has ended. Spills in river systems and pulses mobilized from rain events often create brief exposures shorter than modeled by the traditional 96 h duration and to inform risk assessment we chose to model a 24 h exposure.

Small-bodied freshwater fishes such as minnows of the Leuciscidae family are of growing conservation concern, with global declines driven by invasive species, habitat loss, and pollution^11–15^. As both primary and secondary consumers, small-bodied freshwater fish transfer basal energy from periphyton, detritus, and aquatic invertebrates to higher trophic levels and perform many essential ecosystem functions. Many non-aquatic species depend on healthy aquatic ecosystems, especially in arid regions^16,17^. Native stream fishes are key indicators of environmental health and offer insights into changes in water quality and ecosystem stability^18^.

Small-bodied adult prey fish were exposed to 14.64 mg/L diesel fuel for 24 h to simulate a brief pulse analogous to a diesel spill in a small lotic (stream) system^19^. Predator avoidance was assessed to determine if sublethal levels of petroleum can manifest into mortality. The pathogenesis of decreased predator avoidance was examined by lateral line vital dye studies and histopathology.

## Results

### Diesel exposure impairs predator avoidance

**Fig. 1:**
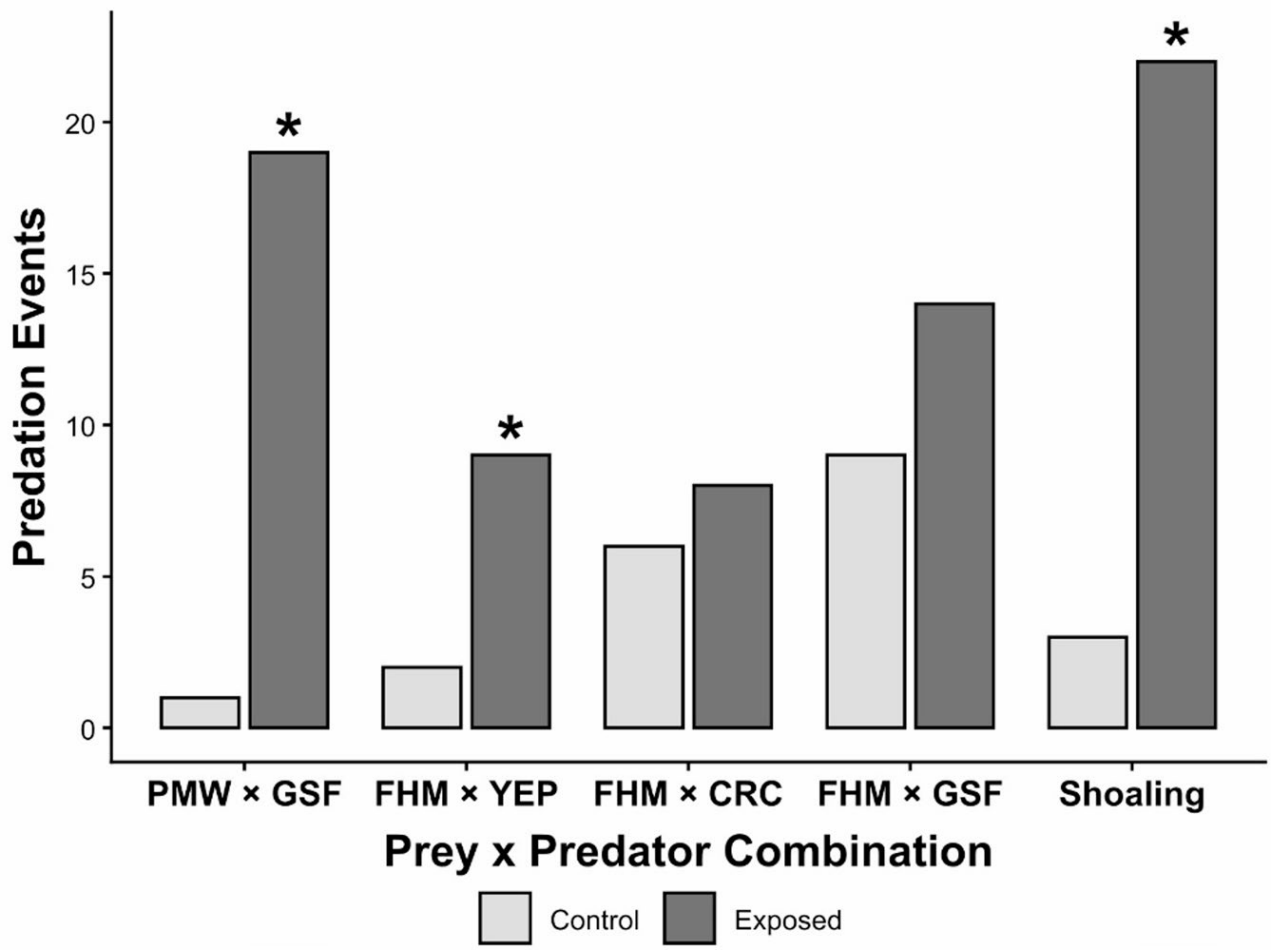
Number of diesel-exposed or control predated fish across four unique prey–predator combinations and shoaling experiments. Full statistical results are presented in Table 1. PMW, Plains Minnow; GSF, Green Sunfish; FHM, Fathead Minnow; YEP, Yellow Perch; CRC, Creek Chub; FHC, Flathead Chub. The asterisks (*) denotes *p* value < 0.05. PMW × GSF *n* = 20 trials; FHM × YEP *n* = 11 trials; FHM × CRC *n* = 14 trials; FHM × GSF *n* = 23 trials; FHC × GSF shoaling *n* = 2 trials; FHM × GSF shoaling *n* = 6 trials.

Prey fish, including Plains Minnow (*Hybognathus placitus*, PMW), Fathead Minnow (*Pimephales promelas*, FHM), and Flathead Chub (*Platygobio gracilis*, FHC), were exposed to 14.64 (± SD 7.65) mg/L diesel across all trials (Supplemental Tables 1 and 2) for 24 h. No mortality was observed. One diesel-exposed prey fish and one control prey fish of the same species were placed in a predator avoidance chamber with a Green Sunfish (*Lepomis cyanellus*, GSF), a Yellow Perch (*Perca flavescens*, YEP), or a Creek Chub (*Semotilus atromaculatus*, CRC). Across all predation events diesel-exposed fish represented 77% of predated fish (Table 1; Fig. 1). For two predator-prey combinations predation of diesel-exposed fish was statistically greater than 50% (no effect). GSF consumed diesel-exposed PMW first in 95% of trials (*p* < 0.0002), and YEP consumed diesel-exposed FHM first in 82% of trials (*p* = 0.0327). This trend was observed in all other predator-prey pairings, although not statistically significant: CRC predated exposed FHM first in 64.3% of trials (*p* = 0.3953) and GSF consumed exposed FHM in 60.9% of trials (*p* = 0.2024).

**Table 1:** Binomial test results of predator avoidance following 24-h diesel-exposure. One tailed *p* values below 0.05 indicate trials where diesel-exposed fish were significantly more likely to be consumed first than expected by chance (50%). PMW, Plains Minnow; GSF, Green Sunfish; FHM, Fathead Minnow; YEP, Yellow Perch; CRC, Creek Chub; FHC, Flathead Chub.

Predator avoidance trials	# of trials	Proportion of trials in which diesel-exposed fish consumed first	95% CI	*p* value (one tailed)
PMW × GSF	20	0.95	0.7512672–0.9987349	0.00002*
FHM × YEP	11	0.818	0.4822441–0.9771688	0.03271*
FHM × CRC	14	0.571	0.2886094–0.8233889	0.3953
FHM × GSF	23	0.609	0.3854190–0.8029236	0.2024
All predator-avoidance trails	68	0.735	0.6142896–0.8349615	6.542e-05*

**Table 2:** Shoaling trials were evaluated with a sign test. One tailed *p* values below 0.05 denote where the median proportion of predation events in which diesel-exposed fish were consumed across all trials was significantly greater than 0.5. FHC, Flathead Chub; GSF, Green Sunfish; FHM, Fathead Minnow.

Shoaling trials	# of trials	Proportion of predation events in each trial that were diesel-exposed fish	95% CI	*p* value (one tailed)
FHC × GSF	2	1, 0.66		0.25
FHM × GSF	6	1, 1, 1, 0.66, 1, 0.66, 1, 0.66	0.66–1	0.0156*
All shoaling trials	8		0.66–1	0.0039*

Coordinated swimming in large groups (also known as shoaling or schooling) is a common predator avoidance tactic. This behavior relies on vision and lateral line sensation^20–23^. Predation trials were also conducted with schools of prey (*n* = 6 or 8; FHC or FHM) and multiple predators (GSF) to determine if the opportunity to swim in groups ameliorated susceptibility of diesel-exposed prey to predation. Trials started with a ratio of 50% control and 50% diesel-exposed minnows. 75% of all FHC and 73% of all FHM predated by GSF in shoaling experiments were diesel-exposed fish. For all shoaling experiments the proportion of diesel-exposed prey consumed was statistically more than 0.5 (*p* = 0.0039). These findings indicate that shoaling behavior is compromised following diesel exposure, which may increase vulnerability to predators and ultimately elevate risk of mortality.

### Diesel exposure decreases viable neuromasts

**Fig. 2:**
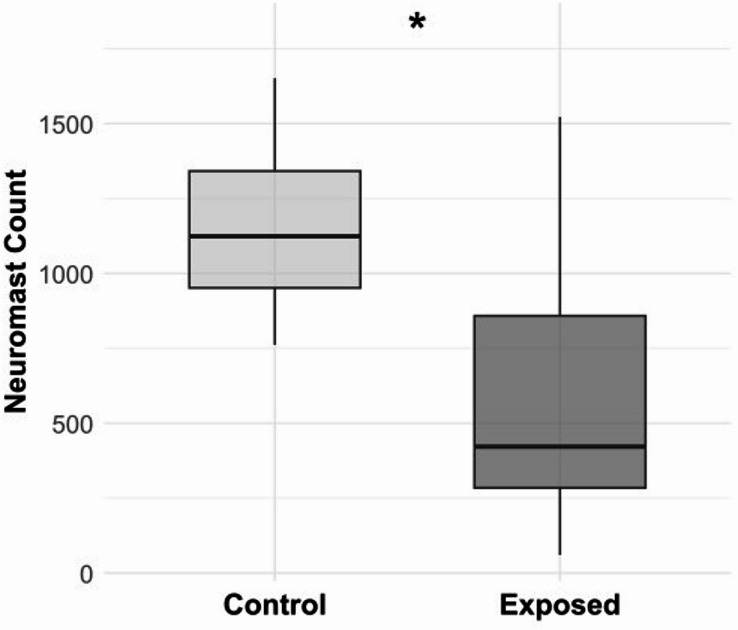
Average whole body lateral line neuromast cell counts of control (*n* = 8) and diesel-exposed Fathead Minnow (*n* = 8) (*Pimephales promelas*). Diesel-exposed Fathead Minnow had significantly fewer neuromasts compared to the control group. Whiskers indicate 1.5 × interquartile range, the boxes represent the 25th–75th percentiles, and the horizontal line inside each box shows the median. Asterisk indicates statistically significant difference between the two groups (*p* < 0.05).

Neuromasts are sensory structures distributed superficially on the skin and in specialized cutaneous canals. They sense changes in local water pressure and flow, such as the bow wave of a predator strike or movement of conspecifics in the shoal and allow fish to reflexively respond to movement. Loss of neuromast function could reduce ability to evade predators and impair shoaling behavior. Vital dye uptake is commonly used to assess the function of neuromast hair cells and is a reliable proxy for mechanotransduction and hair cell integrity^24^. Reduced or absent dye uptake indicates hair cell degeneration, dysfunction, or necrosis and is a useful indicator of sensory impairment^24–26^. Fathead Minnow exposed to diesel for 24 h had markedly diminished numbers of neuromasts (580.25 ± 483.24) detected by vital dye uptake compared to control FHM (1157.75 ± 290.25) (*p* = 0.0074) (Fig. 2, Supplemental Table 6). This was especially true for the tail region, where diesel-exposed FHM had significantly fewer neuromasts (37.8 ± 45.57) than control fish (200.88 ± 49.2) (*p* = 0.0007) (Supplementary Table 2, Supplementary Fig. 1). Diesel-exposed fish also had significantly fewer neuromasts on the face (285.13 ± 190.24) than control fish (489.13 ± 103.803) (*p* = 0.0325) (Supplementary Table 2, Supplementary Fig. 1). Lateral line impacts would go unnoticed in laboratory toxicity experiments that assessed mortality alone, but in nature predator avoidance, shoaling, and other behaviors reliant on this sensory system would be impaired.

**Fig. 3:**
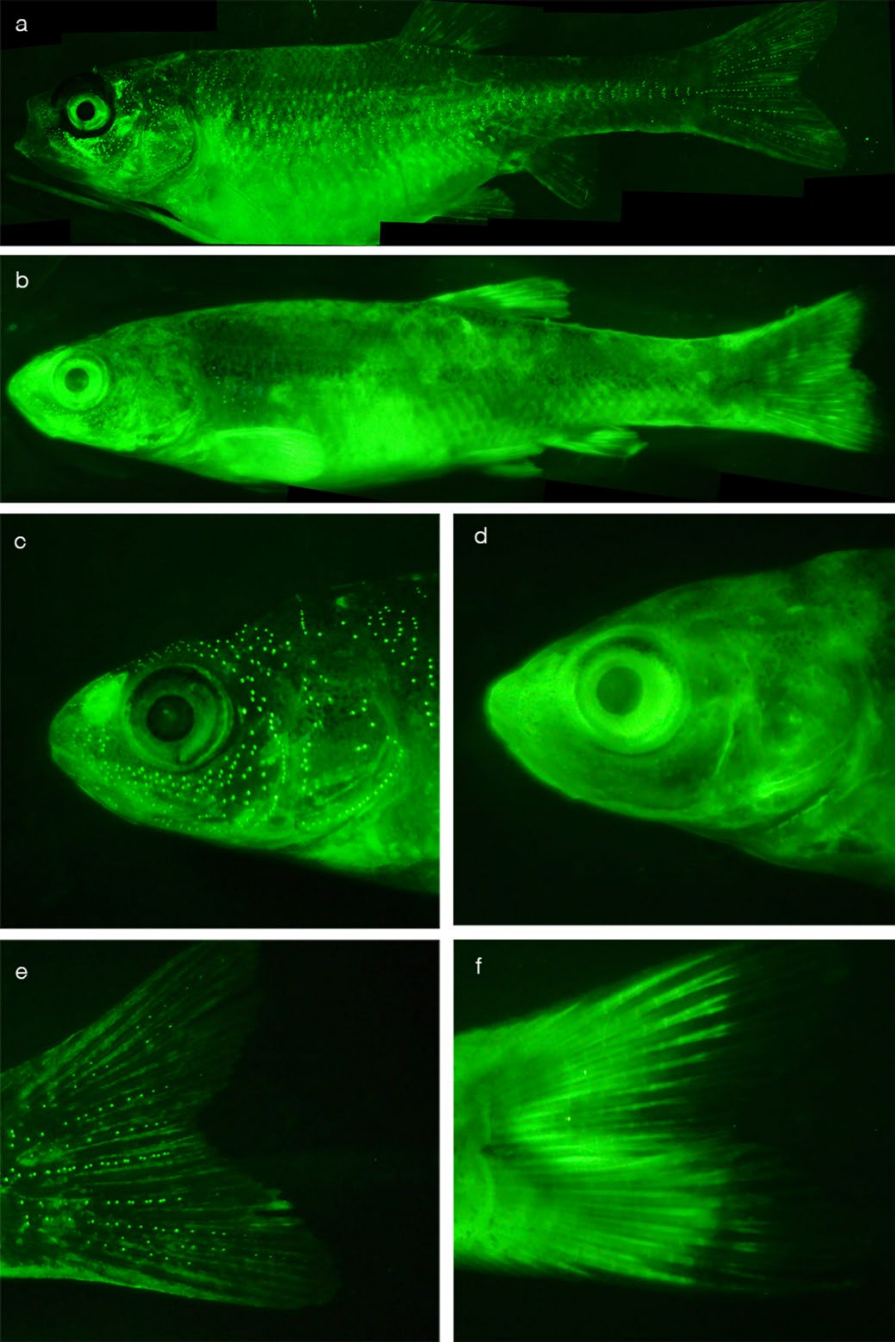
Distribution of neuromasts in control Fathead Minnow (**a**,**c**,**e**), and Fathead Minnow exposed to diesel for 24 h (**b**,**d**,**f**) as detected by fluorescent 4-Di-2-ASP vital dye and imaged with blue light. Bright green foci represent dye uptake by hair cells of neuromasts. Lack of bright green foci indicates decreased neuromast function in exposed fish. Note opacification of the cornea in the diesel-exposed fish (**d**), which corresponds to keratitis and corneal edema.

### Diesel exposure causes ocular and gill pathology

**Fig. 4:**
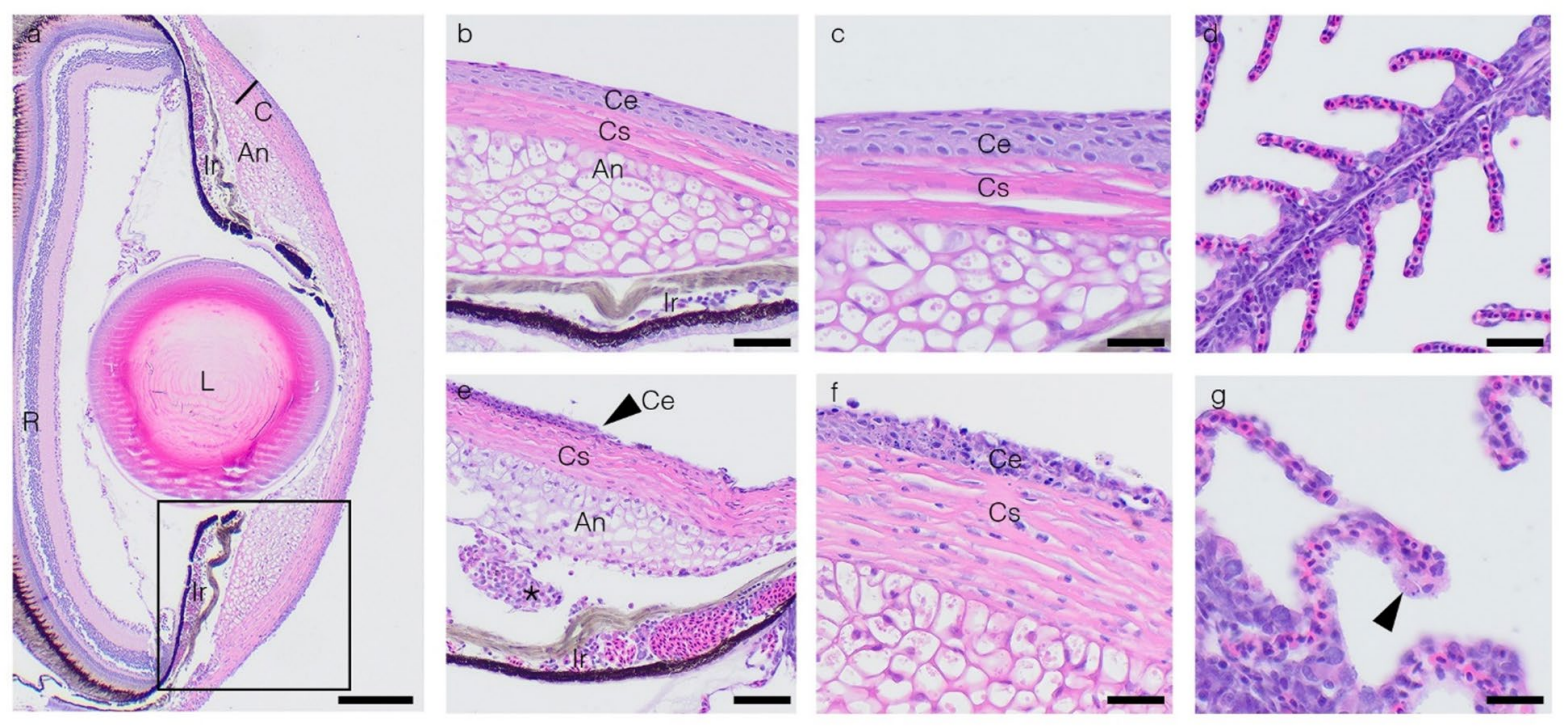
Histopathology of control and diesel-exposed Fathead Minnow. (**a**) Anatomy, control FHM eye. The corneal surface is directed towards the right. C, cornea, An, annular ligament, Ir, iris, L, lens, R, retina. Box indicates the iridocorneal angle. Scale bar = 200 μm. (**b**) Higher magnification of iridocorneal angle from a control FHM as pictured in (a) but rotated 90 degrees counterclockwise; corneal surface is now directed upwards. The cornea is comprised of the corneal epithelium, Ce, and corneal stroma, Cs. An, annular ligament. Scale bar = 50 μm. (**c**) Superficial cornea, control FHM. The corneal epithelium is 4–5 cell layers thick and uniform. Scale bar = 20 μm. (**d**) Gill, control FHM. Secondary lamellae are uniform in thickness and length. Scale bar = 20 μm. (**e**) Iridocorneal angle, diesel-exposed FHM with anterior uveitis and keratitis. The corneal epithelium is attenuated (arrowhead), the corneal stroma is infiltrated by leukocytes, and there are inflammatory cells in the anterior chamber (*, between An and Ir). Scale bar = 50 μm. (**f**) Superficial cornea, diesel-exposed FHM with keratitis. The corneal epithelium is stippled by pyknotic and karyorrhectic nuclear debris. The collagen of the stroma is smudgy with small amounts of nuclear debris and is hypercellular due to infiltrating leukocytes. Scale bar = 20 μm. (**g**) Gill, exposed FHM with focal acute necrosis of branchial epithelium. The necrotic cell has hypereosinophilic cytoplasm and pyknotic/karyorrhectic nucleus (arrowhead). Scale bar = 20 μm.

**Fig. 5:**
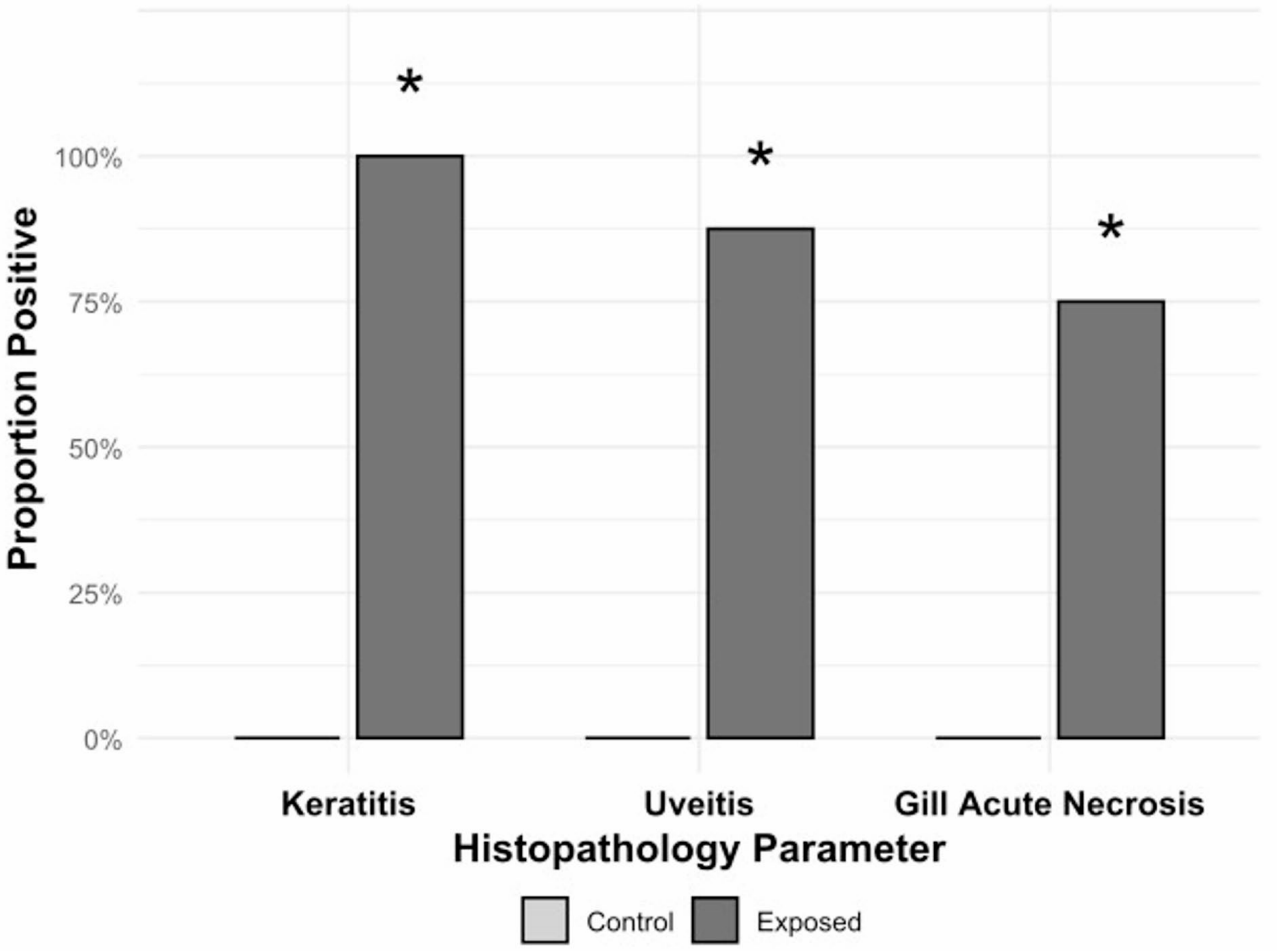
Proportion of control (*n* = 8) and diesel-exposed fish (*n* = 8) with keratitis, uveitis, and acute multifocal gill necrosis. Asterisks indicate statistically significant differences between groups (*p* < 0.05).

Pathology affecting sight, branchial oxygen transfer, and the lateral line system were investigated as a potential explanation for reduced predator avoidance. Figures 4 and 5 summarize key histopathologic findings. Subgross histopathology of a FHM eye is presented in Fig. 4a for orientation. Control fish had a clear, unobstructed iridocorneal angle (Fig. 4b). The corneal epithelium of control fish was 4–5 cell layers thick and comprised of uniform cuboidal to polygonal cells with discrete cell borders, modest amounts of eosinophilic cytoplasm, and round to oval nuclei with homogenous chromatin, and the corneal stroma was comprised of uniform collagen (Fig. 4c). Control gill had normal intact secondary lamellar architecture (Fig. 4d).

Seven of eight diesel-exposed fish had anterior uveitis while no control fish had anterior uveitis (Fig. 5, *p* = 0.0007). In exposed fish there were clusters of lymphocytes and macrophages in the anterior chamber between the cornea and iris or adhered to either the surface of the caudal cornea or anterior iris (Fig. 4e). All exposed fish had marked dilation of iridal vasculature (physiologic hyperemia). All FHM exposed to diesel had acute necrotizing keratitis while no keratitis was identified in control fish (Fig. 4e and f, *p* < 0.0001). The corneal epithelium of seven exposed fish was attenuated to two to three recognizable cell layers and disrupted by necrosis. In exposed fish the corneal stroma was infiltrated by low numbers of leukocytes (Fig. 4d). The eighth exposed fish had diffuse necrosis and complete loss of the epithelium and necrosis of the corneal stroma, which was homogeneously eosinophilic without distinction of collagen fibers. Both keratitis and anterior uveitis would be expected to cloud vision and could contribute to poor predator avoidance. These sublethal findings would not be captured in a study focused solely on mortality.

Six of eight treated fish had multifocal mild acute necrosis of individual epithelial cells lining secondary lamellae (Fig. 4g), which was not identified in any control fish (*p* = 0.0035). Acute branchitis also represents a subclinical finding that would not be identified in a standard toxicology test. Two of eight exposed fish had mild focal branchial telangiectatic change, which was not identified in any control fish, but this was not statistically significant (*p* = 0.4771). Both diesel-exposed and control fish had the same degree of interlamellar filling by proliferative epithelial cells (mild to moderate proliferative branchitis) (*p* = 0.5501, considered a background lesion). Rare gill-associated monogenean parasite profiles were identified in both groups (50% of control, 40% of exposed, *p* = 1.0).

An average of 5.3 superficial neuromasts were detected histologically per control fish (median 5 per fish, range 3–9). An average of 7.3 superficial neuromasts were detected histologically per diesel-exposed fish (median 5 per fish, range 2–14). Two of seven control FHM with greater than two superficial neuromasts identified in histologic sections exhibited apoptosis within superficial neuromasts while five of six exposed FHM with greater than 2 superficial neuromasts in histologic section had this change (*p* = 0.1026). Canal neuromasts were encountered so infrequently in histologic section that these were not sufficient for evaluation.

## Discussion

Prey fish exposed to sublethal diesel concentrations were more likely to be predated upon than non-exposed prey fish and this trend was consistent among all trials. Even when provided the opportunity for defensive shoaling diesel-exposed prey were eaten at a higher rate. Thus, failure to avoid predation was more sensitive than mortality caused by diesel exposure alone.

FHM exposed to sublethal diesel had significantly decreased functional neuromasts and significantly increased prevalence of acute severe keratitis and anterior uveitis and branchitis. Successful predator avoidance and shoaling activity depends on the lateral line sensation and vision^20–23^. Damage to the neuromasts and eyes (keratitis and uveitis) was only seen in diesel-exposed fish. It is highly probable that this caused the reduced predator evasion measured in this study. Neuromasts of larval Zebrafish are highly sensitive to crude oil exposure^27^ and blocking lateral line function with other toxicants impairs larval predator escape response^23,28^. Our findings strongly suggest that diesel-induced lateral line damage impairs predator evasion of adult minnows. While crude oil is known to cause retinal apoptosis and disrupt ocular development in larval fish with implications for long term vision^29,30^, we believe this is the first report of keratitis and anterior uveitis secondary to diesel exposure in adult fish. Mild acute gill necrosis may be due to oxidative injury or direct membrane effects, but is of uncertain significance due to the capacity of this tissue for rapid regeneration^31^. Gill necrosis may become more important in chronic exposures or at higher concentration. Importantly, cardiac toxicity has been reported in larval fish^32,33^ and cardiac explants of adult fish^34,35^ exposed to PAHs but was not assessed in our study. Reduced cardiac performance could lead to reduced swim performance and failure to evade predators, especially during aerobic swimming of long duration. Future work should investigate the propensity for systemic absorption and internal tissue distributions of various PAHs and should include functional cardiac endpoints to determine the potential contribution of cardiac toxicity following acute PAH exposure.

Loss of predator avoidance after exposure to diesel was observed in all three prey minnow species tested and provides initial insights into the sensitivity of the Plains Minnow and Flathead Chub, for which no prior data exists. The Leuciscidae family of freshwater minnows represents nearly one-third of all freshwater fish species currently at risk of extinction globally from impacts such as habitat destruction and fragmentation, anthropogenic water use, pollution, and the spread of nonnative predators or invasive species^13^. These fish fill critical niches in freshwater ecosystems. Their abundance and trophic position make them key forage species within food webs across freshwater and riparian ecosystems, thus linking aquatic production to terrestrial consumers such as birds and mammals^16,18^. Little literature is available for native minnows in our area, where tanker trailers, car accidents, and trail derailments regularly result in brief (often < 24 h) pulses of diesel spills into rivers. We suspect the diesel susceptibility we identified may be conserved across all small-bodied fish species and all young age classes of large-bodied fish species.

The average measured diesel concentration in our 24-h study was 14.6 mg/L TPH of DRO (Supplemental Table 2). After an initial loading rate of 75 mg/L diesel fuel water concentrations were measured at 23.9 mg/L in the first minutes of exposure and 7.3 mg/L at the end of the 24 h exposure. We identified loss of predator avoidance and numerous pathologies at concentrations below acute LC_50_ values (concentration lethal to 50% of test organisms after 96 h). Acute 96 h)sensitivity of fish species to diesel fuel are highly variable with LC_50_ values that range from below 5 mg/L to over 100,000 mg/L (Supplemental Table 5). For adult FHM, a key species in our study, LC_50_ for acute 96 h diesel exposures range from 48 to >160 mg/L based on loading rate^6,36,37^. LC_50_ values for salmonids (*Oncorhynchus* spp.) exposed to diesel for 24 h range from 1,404 to > 168,363 mg/L^38–41^. It can be normal to see modest differences in reported sensitivity between species and age classes. However, discrepancies in reporting and experimental methods are a possible explanation for these remarkably wide ranges. Some studies only report nominal or loading rates while others report measured chemical concentrations^37^. LC_50_ estimates calculated using loading rates or nominal values will be overestimated due to rapid toxicant loss. Methods that include extensive emulsification and flow through exposure found LC_50_ values as low as ~0.032 mg/L for FHM, suggesting environmental realism could be improved in all studies including ours^42^. Studies that vigorously emulsify the diesel prevent hydrocarbons from partitioning to surfaces and forcibly disperse the dose into solution. The sun’s ultraviolet (UV) radiation can photo-excite hydrocarbons in diesel inducing photo-enhanced toxicity^43,44^, yet this mechanism is often overlooked. Studies that included natural substrate observed a rapid loss of toxicants in the water column but can better model how a pulse exposure event can become a press exposure event as fuels are bound to habitat^19^. These discrepancies make it extremely challenging to incorporate data in risk assessment. We exposed fish to diesel fuel rather than individual chemical components in order to model realistic diesel spill events. UV lamps were employed to provide photoexcitation, though not at the intensity possible with natural sunlight. Loading rate and observed concentrations are both important to report because first responders can often estimate the volume of fuel spilled and the most common chemical assessment of surface waters is TPH of DRO.

Neuromasts are very small structures (~100 microns in diameter) and can be difficult to capture histologically. The low number of neuromasts in histologic section may have prevented recognition of significant differences in neuromast histopathology. Future studies should consider ultrastructural evaluation by scanning electron microscopy to examine acute pathology of neuromasts. Neuromasts are known to regenerate rapidly following some toxicant exposures^45–47^, and greater work is needed to assess the potential for neuromast regeneration following diesel exposure. Further studies should also evaluate the potential for resolution or progression of branchitis and ophthalmic pathology, including the potential for chronic corneal scarring and complications of chronic anterior uveitis such as glaucoma. Future work is also needed to discern if brief sublethal exposures may result in direct mortality unrelated to predation pressures.

Fish conservation relies on the creation of protective standards which in turn requires scientists to consider sublethal effects that are likely to manifest into population loss. These new behavioral and histopathologicl endpoints have potential to improve toxicity experiments. Decades of acute aquatic toxicology studies have focused on mortality, the most obvious response variable. This can upwardly bias the reported mean toxic threshold concentrations (such as LC_50_ and EC_50_). A chief way to improve the realism of toxic thresholds is to incorporate sublethal effects. With only one treatment level our study is unable to estimate effect concentrations, yet the methods to characterize predator avoidance and novel toxicologic endpoints presented here have the potential to markedly improve the environmental relevance of toxicity experiments used to derive pollutant limits for the numerous individual components of diesel fuel. Application of these methods to sensitive species and early life stages in 96 h and > 30 d exposures will likely result in lower more environmentally sound estimates of toxic thresholds.

## Materials and methods

### Test organisms

Prey species from the Leuciscidae family included Plains Minnow (*Hybognathus placitus*, PMW), a species listed as endangered in Colorado; the Flathead Chub (*Platygobio gracilis*, FHC), a Colorado species of concern; and the Fathead Minnow (*Pimephales promelas*, FHM), a species widely distributed across North America and commonly used in aquatic toxicological tests^48,49^. Plains Minnow (mean total length [MTL ± SD] 40 mm ± 12) were obtained from the Colorado Parks and Wildlife Native Aquatic Species Restoration Facility (Alamosa, CO, USA), FHM (MTL 35 mm ± 3) were sourced from Aquatic Bio Systems (Fort Collins, CO, USA), and FHC (MTL 44 mm ± 15) were collected via backpack electrofishing from Fountain Creek in Colorado Springs, CO, USA. Predator species included native Green Sunfish (*Lepomis cyanellus*, GSF; MTL 160 ± 38 mm) collected by trap net from Running Deer Open Space (Fort Collins, CO, USA), introduced Yellow Perch (*Perca flavescens*, YEP; MTL 200 mm ± 20) captured by hook and line from Seaman Reservoir (Larimer County, CO, USA), and native Creek Chub (*Semotilus atromaculatus*, CRC; MTL 170 mm ± 25) obtained by backpack electrofishing from the South Platte River near Sterling, CO, USA. All collections and methods were approved by Colorado Parks and Wildlife institutional IACUC for Aquatic Research, were conducted in accordance with relevant state and federal regulations and institutional directives, and complied with ARRIVE and IUCN guidelines.

### Diesel exposures

Prey fish used in predator avoidance studies were marked intradermally with ultraviolet fluorescing blue, red, orange, or green Visual Implant Elastomer (VIE, Northwest Marine Technology, Ancotes, WA, USA) along the dorsal fin, and allowed to recover for ≥ 14 d. Stainless steel aquaria (6 L, Polarware, Sheboygan, WI, USA, Model G12066) were filled with 3,000 mL of dechlorinated municipal tap water (Fort Collins, CO, USA). Control (0 mg/L) and treatment (14.64 ± 7.65 mg/L) concentrations were created by adding the appropriate aliquot of diesel fuel (Schrader Oil Co., Fort Collins, CO, USA, Grade No. 2-D S15, ASTM D975) in water at a loading rate of 0 mg/L or 75 mg/L. This concentration was found to result in no mortality for salmonids after 96 h exposure in the same experimental system^19^. Fish were randomly assigned to the aquaria. A temperature-controlled water bath held all aquaria at 17.26 (± 1.1)°C. Sodium silicate Pasteur pipettes attached to a blower delivered aeration. Diesel fuel is potentially more bioavailable when photoexcited by the UVA + B spectra (UVA:320–400 nm UVB:290–320 nm) common in natural sunlight^43,44,50^. Wide spectrum light bulbs (ZooMed Laboratories, Reptisun 10.0 UVB, F32T8/REP, San Luis Obispo, CA, USA) with UVA + UVB were hung 15 cm over the aquaria to simulate natural outdoor light at a 16:8 h light: dark photoperiod. These provided ultraviolet light (UV-A + B) intensity of 1.5 mW/cm^2^ at the surface of the water (as measured by UVA/B Meter Model:850009, Sper Scientific, Scottsdale, AZ, USA), which is relatively one tenth of what would be observed with direct sunlight (14.5 mW/cm^2^). After the 24 h exposure prey fish were placed in identical aquaria (6 L hotel pans) with fresh, uncontaminated water for at least fifteen minutes before assessing predator avoidance.

### Diesel concentrations and water quality

Water samples from a randomly assigned subsample of replicate tanks were taken from the middle of the water column for assessment of TPH. Samples were sent to a state certified analytical chemistry laboratory (ACZ Laboratories, Inc., Steamboat Springs, CO, USA) for analysis of extractable diesel fuel range organics (DRO) total petroleum hydrocarbons C10-C28 (TPH) using the Environmental Protection Agency’s (EPA) M8015D/API-DRO GC/FID method. Samples from the 24 h exposure tanks showed average observed levels of TPH at 14.64 ± 7.65 mg/L in the water column (Supplemental Table 1). Treatment replicates were sampled at the beginning (0 h), middle (12 h), or end (24 h) of each exposure to characterize loss of toxicant (Supplemental Table 2). It is common to lose a majority of diesel fuel from the water column due to volatilization, coalescence and partitioning to the water surface, and adherence to container walls during toxicity trials^19,51^. No trace diesel was observed in controls or in predator avoidance test tanks (minimum detection limit of 0.25 mg/L). Water chemistry data (temperature, pH, dissolved oxygen, hardness and alkalinity) was collected from all exposure and control tanks across all experiments. Dissolved oxygen, temperature, alkalinity, hardness, and pH remained consistent and at levels ideal for fish culture across all exposure and control aquaria (Supplemental Table 1).

### Predator avoidance tests

Predator-prey combinations were selected to represent realistic predatory–prey interactions in eastern Colorado based on 30 years of state biomonitoring records (Supplemental Table 3). Predator species were not exposed to diesel and were fasted for 24 h prior to trials. Diesel-exposed and control prey of similar mean total length (MTL; <0.45 mm difference) were paired with a large-bodied predator. For each combination prey fish MTL were within 23.5% (0.055 SD) of the predator MTL. This is well below the maximum prey size recommended of 33%^52^. All VIE tag colors were evenly distributed across trials to ensure no single color was associated with exposed or control fish more frequently.

The primary focus of this study was on trials that consisted of one randomly selected diesel-exposed prey, one randomly selected control prey, and one predator (2–23 trials per prey-predator species combination). Fish were randomly selected and placed into a circular black plastic tank (204 L; KMB 101, Tuff Stuff Products, Terra Bella, CA, USA) filled to 33% of tank depth. For 15–30 min, the prey fish were confined within a mesh cylinder that protected them from the predator. After this acclimation period, the mesh barrier was removed, allowing fish to interact freely. Each trial continued until one of the two prey fish was consumed. Trial duration ranged from 36 s to 17.6 h (mean 2.07 h ± 4.6 SD). The surviving prey fish was then removed from the trial chamber to verify treatment based on tag color. Following predator evasion trials all fish were euthanized using buffered tricaine methanesulfonate (MS-222) and length (mm) and weight (grams) were determined.

Abnormal shoaling behavior was incidentally noted among diesel-exposed fish. To explore this, a smaller number of shoaling trials were also conducted, in which three or four fish from the diesel-exposed prey and control prey groups (FHC or FHM) were introduced alongside two, three, or four predators (GSF). Shoaling trials were conducted in the same tank system after a 15–30-min acclimation period during which the prey species were protected by mesh. Shoaling trials were terminated when 50% of the total prey was consumed or when 3 h had elapsed at which time remaining fish were removed to verify identification based on tag color followed by euthanasia with buffered MS-222 and determination of length (mm) and weight (grams). The total number of diesel-exposed and control fish consumed per trial was recorded.

Statistical analysis of predator-prey trial data was conducted using binomial tests in R 4.5.2 to test the a priori directional hypothesis that exposure to diesel would increase the probability of mortality in diesel-exposed fish relative to control fish. Sign tests were applied to shoaling trials to compare the a priori directional hypothesis that the median proportion of diesel-exposed fish consumed in each trial was greater than 0.5.

### Vital dye staining

Vital dye analysis has been used to assess morphology of the lateral line, effect of climate change on lateral line development^53^, and the effect of numerous toxicants^24,25^. An individual FHM from each replicate (*n* = 8 controls and *n *= 8 exposed) was placed in 0.0024% 4-Di-2-Asp (4-(4-Diethylaminostyryl)-*N*-methylpyridinium iodide, 4-Di-2-ASP, VWR, 102989-616) solution for five minutes. Separate dye containers were used for control fish and diesel fish to avoid diesel contamination. Fish were rinsed in fresh system water then euthanized via immersion in buffered MS-222. Fluorescence of hair cells in superficial and canal neuromasts was imaged with a MEIJI EMZ-TR dissecting microscope on a boom stand in a darkroom. The objective lens was adapted with a royal blue-green (550–560 nm band pass) filter. A royal blue light (440–460 nm) was used to illuminate target areas (SFA-RB-GO, NightSea, Inc., Hatfield, PA, USA). Gain and exposure time were held constant throughout all imaging. Images and video of both sides of each fish were captured with a Unitron Excelis HD color digital camera and CaptaVision+ software (Accu-scope, Inc, Commack, NY, USA). Serial images of fish were stitched together with AutoStitch to produce images of the whole left and right sides^54^. Total neuromast counts from each fish (left + right sides) were assessed by Cell Profiler (V3)^55^ with manual quality control. A subset of each body was reassessed (left + right) to capture just the caudal fin rays (caudal to the peduncle) and cranial region (snout to insertion of the pectoral fin) as these regions were predicted to respond differently. Diesel-exposed and control fish neuromast counts (total body, total tail, and total head) were compared with a Wilcoxon rank-sum test due to the small sample size and potential non-normal distribution of the data. As there were no significant differences in length or weight between diesel-exposed and control fish, these variables were not included as covariates in the statistical analyses (Supplemental Table 4).

### Histopathology

FHM from the vital-dye studies (*n* = 8 controls and *n* = 8 exposed) were immersed whole in 10% neutral buffered formalin for > 48 h. Fish were trimmed coronally (i.e., through the transverse plane) through the middle of the eyes, branchial chamber, and the body to represent viscera (including heart, liver, intestine, kidney, spleen, body wall). Trimmed specimens were processed routinely to 5 μm thick microscope slide sections stained with hematoxylin and eosin. Fish were scored for the presence or absence of acute keratitis, anterior uveitis, and gill necrosis and were compared with Fisher’s exact tests. All sections of available globe (up to 4 per fish) were included in analysis. All available primary and secondary lamellae were included in analysis. The degree of interlamellar filling was scored as absent, mild (less than ⅓ of the interlamellar space occluded), moderate (⅓ to ⅔ occluded), or severe (>⅔ occluded). The degree of interlamellar filling was compared by Wilcoxon rank-sum test. The incidental presence or absence of rare monogenean branchial parasites was noted and compared by Fisher’s exact test. The presence or absence of apoptosis in superficial neuromasts was recorded and compared by Fisher’s exact test. Only fish with 2 or more superficial neuromasts histologically represented were included in the evaluation.

## Supplementary Information

Below is the link to the electronic supplementary material.

Supplementary Material 1

## Data Availability

The authors declare that data supporting the findings of this study are available within the paper and its supplementary information files.
