# Supplementary material for: Reduced predator avoidance follows ocular and lateral line pathology in minnow species after diesel exposure
Source: Sci Rep. 2026 May 26;16:23975. doi: 10.1038/s41598-026-53051-z (PMC13434130; doi:10.1038/s41598-026-53051-z)
Supplement: Supplementary file 1 [file 41598_2026_53051_MOESM1_ESM.pdf]

Supplemental Materials

Supplemental Table 1. Water Chemistry. Total Petroleum Hydrocarbons (TPH) of extractable Diesel Range Organics (DRO), temperature, dissolved oxygen (DO), hardness, alkalinity, and pH. ND = Not Detected (below minimum detection limit of 0.235 mg/L).

Parameter	Control (mean +/- SD)	Exposed (mean +/- SD)
Diesel Loading Rate mg/L	0	75
Average Measured Diesel Concentration (TPH C10 to C28 mg/L)	ND<br>n=18	14.64 +/- 7.65<br>n=33
Average Temperature (Celsius), n=32	17.19 +/- 1.07	17.21 +/- 1.15
Average DO mg/L, n=9	9.34 +/- 0.16	9.21 +/- 0.16
Average Hardness mg/L $CaCO_3$, n=17	55.41 +/- 4.38	55.72 +/- 3.52
Average Alkalinity mg/L $CaCO_3$, n=17	41.11 +/- 6.72	40.59 +/-5.15
Average pH, n=9	7.69 +/- 0.08	7.64 +/- 0.11

Supplemental Table 2. Average measured concentrations of diesel in the water column measured 0, 12, and 24 hours after loading 75 mg/L diesel.

	0 hours	12 hours	24 hours
Average +/- SD Measured Diesel<br>Concentration (TPH C10 to C28 mg/L)	23.9 +/- 3.2<br>n = 11	12.8 +/- 2.8<br>n = 11	7.3 +/- 0.9<br>n = 11

Supplemental Table 3. Colorado Parks and Wildlife (CPW) sampling records for predator and prey species.

**Prey**	**Predator**	**Years of Co-occurrence Observed**	**Total Number of Co-occurrence Bio-monitoring events**
Plains Minnow (PMW, *Hybognathus placitus*)	Green Sunfish (GSF, *Lepomis cyanellus*)	1950-2023	31
Fathead Minnow (FHM, *Pimephales promelas*)	Yellow Perch (YEP, *Perca flavescens*)	1912-2023	503
Fathead Minnow (FHM, *Pimephales promelas*)	Creek Chub (CRC, *Semotilus atromaculatus*)	876-2023	3117
Fathead Minnow (FHM, *Pimephales promelas*)	Green Sunfish (GSF, *Lepomis cyanellus*)	1876-2023	3117
Flathead Chub (FHC, *Platygobio gracilis*)	Green Sunfish (GSF, *Lepomis cyanellus*)	1889-2023	197
Fathead Minnow (FHM, *Pimephales promelas*)	Green Sunfish (GSF, *Lepomis cyanellus*)	1903-2023	3117

GSF co-occurs with PMW across both the Arkansas River, Republican River, and the South Platte River drainages as recorded by 31 sampling events within three different locations over the past 71 years (internal data, Colorado Parks and Wildlife). Routine state biomonitoring has observed FHM co-occurring with GSF, YEP, and CRC in lotic and lentic systems including the Arkansas, Republican, and South Platte drainages in the past 120 years. The FHC co-occurs in nature with GSF within the Arkansas River drainage.

Supplemental Table 4. Weight and total length of FHM used in vital dye and histopathology studies.

	Control FHM	Diesel-exposed FHM
Average total length (mm, SD)	37.125 (+/-3.136)	33.375 (+/-5.655)
Average weight (g, SD)	0.55 (+/- 0.146)	0.4475 (+/-0.164)

There was no statistical difference in total length of fish (Mann-Whitney Test for Two Independent Samples. Control: Count=8 Rank Sum=83 U=17 Exposed: Count=8 Rank Sum=53 U=47. p =0.130 with exact variance) or fish mass (Mann-Whitney Test for Two Independent Samples. Control: Count=8 Rank Sum=81 U=19 Exposed: Count=8 Rank Sum=55 U=45. p =0.195 with exact variance).

Supplemental Table 5. LC_{50} values for representative freshwater fish species including exposure duration, test type, and reference.

Species	Method details	Reference
Fathead minnow<br>(*Pimephales promelas)*	LS = adult; W = soft; D = Diesel No 2.<br>flow-through, emulsion<br>96 hr LC_{50} = 0.0386 μL/L<br>E = LR	Hedtke and Puglisi 1982
Fathead minnow<br>(*Pimephales promelas)*	LS = 30-60d; W = soft; D = Diesel No 2.<br>Floating layer, static<br>96hr LC_{50} = 48.3 μL/L<br>E = LR	Hedtke and Puglisi. 1982
Fathead minnow<br>(*Pimephales promelas)*	LS = NR; W = NR; D = Diesel No 2.<br>Flow through<br>96 hr LC_{50} = 57<br>E = NS	Delek US. 2022
Fathead minnow<br>(*Pimephales promelas)*	LS = 30-60d; W = soft; D = Diesel No 2.<br>Floating layer, static<br>96hr LC_{50} >160 μL/L<br>E = LR	Hedtke and Puglisi 1982
Fathead minnow<br>(*Pimephales promelas)*	LS = <48hr; W = soft;<br>Full spectrum light, UV NR<br>D = Fresh Diesel No 2, Static renewal<br>7d LC_{50} = 1.87<br>D = Weathered Diesel No 2, Static renewal<br>7d LC_{50} >4.33<br>E = MC	Hobbs et al. 2018
Pink Salmon<br>(*Onchorhychus gorbuscha)*	LS = juvenile; W = soft; D = Diesel No 2,<br>oil-water mixture, static<br>24 hr LC_{50} = 1972<br>48 hr LC_{50} = 276<br>72 hr LC_{50} = 84<br>96 hr LC_{50} = 74<br>E = LR	Wan et al. 1990
Pink Salmon<br>(*Onchorhychus gorbuscha)*	LS = juvenile; W = intermediate;<br>D = Diesel No 2, oil-water mixture, static<br>24 hr LC_{50} = 1829<br>48 hr LC_{50} = 376<br>72 hr LC_{50} = 133	Wan et al. 1990

	96 hr LC_{50} = 123<br>E = LR	
Pink Salmon (*Onchorhychus gorbuscha)*	LS = juvenile; W = hard;<br>D = Diesel No 2, oil-water mixture, static<br>24 hr LC_{50} = 1404<br>48 hr LC_{50} = 302<br>72 hr LC_{50} = 48<br>96 hr LC_{50} = 32<br>E = LR	Wan et al. 1990
Coho Salmon *(Onchohynchus kisutch)*	LS = juvenile; W = soft;<br>D = Diesel No 2, emulsion, static<br>24 hr LC_{50} = 28972<br>48 hr LC_{50} = 28972<br>72 hr LC_{50} = 12345<br>96 hr LC_{50} = 10299<br>E = LR	Wan et al. 1990
Coho Salmon *(Onchohynchus kisutch)*	LS = juvenile; W = intermediate;<br>D = Diesel No 2, emulsion, static<br>24 hr LC_{50} = >55560<br>48 hr LC_{50} = 55560<br>72 hr LC_{50} = 33216<br>96 hr LC_{50} = 33216<br>E = LR	Wan et al. 1990
Coho Salmon *(Onchohynchus kisutch)*	LS = juvenile; W = hard;<br>D = Diesel No 2, emulsion, static<br>24 hr LC_{50} = >26743<br>48 hr LC_{50} = 26743<br>72 hr LC_{50} = 4845<br>96 hr LC_{50} = 3333<br>E = LR	Wan et al. 1990
Rainbow Trout (*Oncorhynchus mykiss)*	LS = juvenile; W = hard;<br>D = Diesel No 2, emulsion, static<br>24 hr LC_{50} = >3200<br>48 hr LC_{50} = 5525<br>72 hr LC_{50} = 4600<br>96 hr LC_{50} = 3017<br>E = LR	Wan et al. 1990
Rainbow Trout (*Oncorhynchus mykiss)*	LS = juvenile; W = soft;<br>D = Diesel No 2, static<br>24 hr LC_{50} = >3200<br>48 hr LC_{50} = 5525	Wan et al. 1990

	72 hr LC_{50} = 4600<br>96 hr LC_{50} = 3017<br>E = LR	
Rainbow Trout (*Oncorhynchus mykiss)*	LS = juvenile; W = intermediate;<br>D = Diesel No 2.<br>24 hr LC_{50} = >168363<br>48 hr LC_{50} = 168363<br>72 hr LC_{50} = 28787<br>96 hr LC_{50} = 2186<br>E = LR	Wan et al. 1990
Rainbow Trout (*Oncorhynchus mykiss)*	LS = juvenile; W = hard;<br>D = Diesel No 2.<br>24 hr LC_{50} = >23108<br>48 hr LC_{50} = 23108<br>72 hr LC_{50} = 5102<br>96 hr LC_{50} = 2447<br>E = LR	Wan et al. 1990
Rainbow Trout (*Oncorhynchus mykiss)*	LS = NR; W = NR;<br>D = Diesel No 2, semistatic<br>96 hr LL_{50} = 21<br>E = NS	Chevron Phillips Chemical 2024
Rainbow Trout (*Oncorhynchus mykiss)*	LS = Fry; W = NR; D = NR<br>Static renewal<br>14 d EC_{50} = 44.8<br>E = nominal (subclasses of chemicals well characterized)	Mos et al. 2008
Rainbow Trout (*Oncorhynchus mykiss)*	LS =larval; W = NR; D = water soluble fraction of diesel, specific subtype NR; static with agitation<br>48 hr LC_{50} = 2.43-2.52<br>E = MC	Lockhart et al. 1987
Rainbow Trout (*Oncorhynchus mykiss)*	LS = NR; W = NR; D = Diesel No 2<br>96 hr LL_{50} = 65<br>E = NS	Valero Marketing & Supply Company 2014
American shad (*Alosa sapidissima*)	LS = juvenile; W = NR;<br>D = Diesel No 2; static<br>24 hr TLM (median tolerance limit) = 204<br>48 hr TLM = 167<br>E = LR	Tagatz 1961

Blue Gill (*Lepomis macrochirus)*	LS = NR; W = NR; D = NR<br>96 hr LC_{50} = 2.2<br>E = NS	Holly Frontier Refining and Marketing LLC (2022)
Murray River Rainbowfish (*Melanotaenia fluviatilis*)	LS = larvae; W = NR; D = NR<br>96 hr LC_{50} = 0.0213<br>E = NS	Holly Frontier Refining and Marketing LLC (2022)
Banded Killifish (*Fundulus diaphanus*)	LS = NR; W = moderate;<br>D = Diesel No 2, static.<br>96 hr LC_{50} = 26.1<br>E = LR	Rehwoldt et al. 1974
Banded Killifish (*Fundulus diaphanus*)	LS = NR; W = moderate;<br>D = Diesel No 4, static.<br>96 hr LC_{50} = 21.0<br>E = LR	Rehwoldt et al. 1974
Striped Bass (*Roccus saxatilis*)	LS = NR; W = moderate;<br>D = Diesel No 2, static.<br>96 hr LC_{50} = 22.2<br>E = LR	Rehwoldt et al. 1974
Common Carp (*Cyrinus carpio)*	LS = NR; W = moderate;<br>D = Diesel No 2, static.<br>96 hr LC_{50} = 49.1<br>E = LR	Rehwoldt et al. 1974
Common Carp (*Cyrinus carpio)*	LS = NR; W = moderate;<br>D = Diesel No 4, static.<br>96 hr LC_{50} = 48.1<br>E = LR	Rehwoldt, et al. 1974
Common Carp (*Cyrinus carpio)*	LS = NR; W = NR; D = water soluble fraction of diesel, specific type NR, static<br>24 hr LC_{50} = 0.005993%<br>48 hr LC_{50} = 0.005972%<br>72 hr LC_{50} = 0.005942%<br>96 hr LC_{50} = 0.005931%<br>E = LR	Hameed & Al-Azawi 2016
Mud skipper (*Periophthalmus koelreuteri)*	LS = adult; W = brackish; D = water soluble fraction of diesel, specific type NR;<br>*included substrate: mud, mangrove stems, and leaves.<br>96 hr LC_{50} = 4.58 ppm	Bob-Manuel 2012

	E = LR	
Flag Fish (*Jordanella floridae*)	LS = NR; W = soft; D = NR<br>96 hr LC_{50} = 0.0605<br>E = LR	Hedtke and Puglisi 1982
Nile tilapia (*Oreochromis niloticus)*	LS = fingerling; W = NR; D = NR<br>96 hr LC_{50} = 8.08<br>E = LR	Dede and Kaglo 2001
African sharptooth catfish *(Clarias gariepinus)*	LS = fingerling; W = NR; D = NR<br>96 hr LC_{50} = 30<br>E = LR	Ekanem et al. 2018
Golden orfe (*Leuciscus idus melanotus)*	LS = NR; W = NR; D = Diesel No 2, static.<br>96 hr LC_{50} = 120-160<br>E = NS	Juhnke & Lüdemann 1978

Data illustrate the wide range of reported species-specific sensitivities to diesel fuels and wide variation in experimental protocols some of which were intended for standard building and some for hazard response or restoration. This is not a complete table. LS = life stage; W = water type (soft, intermediate, hard); D = specific type of diesel, if reported. All concentrations are in mg/L or as reported by original source. E = indicates whether the effect concentration(s) are based on either measured concentrations (MC), loading rates (LR), or indiscernible/not specified (NS). Toxic thresholds were estimated based on loading rate for many papers and are thus likely to be overestimates. None of these articles include reference to ultraviolet light, which is known to photoexcite diesel components and enhance toxicity. One article included substrate (*Bob-Manuel 2012 included mud, stems, and leaves collected from the environment where the fish were collected). References for this table are listed below.

Hedtke, S. F. & Puglisi, F. A. Short-term toxicity of five oils to four freshwater species. *Arch. Environ. Contam. Toxicol.* **11**, 425–430 (1982). https://doi.org/10.1007/BF01056068

Delek US. Diesel Fuels Safety Data Sheet. (2022). https://s204.q4cdn.com/601669363/files/doc_downloads/sdss/Diesel_Fuels_SDS_NA2015_072122_FINAL1.pdf

Hobbs W.O., Eickoff C. V., and Lee K. Vol. Environmental effects-based concentrations for weathered diesel-range organics toxicity in marine water and freshwater. 20-03-008 (Washington State Department of Ecology, Olympia., 2020). https://apps.ecology.wa.gov/publications/summarypages/2003008.html.

Wan, M. T., Watts, R. G. & Moul, D. J. Acute toxicity to juvenile Pacific salmonids and rainbow trout of butoxyethyl esters of 2,4-D, 2,4-DP and their formulated product: Weedone CB and its carrier. *Bull. Environ. Contam. Toxicol.* **45**, 604–611 (1990). https://doi.org/10.1007/BF01700635

Chevron Philips Chemical Company, Diesel No. 2 Test Fuel: Safety Data Sheet (SDS No. 100000013879). Section 12: Ecological Information. (2024). https://www.cpchem.com/sites/default/files/2024-06/02041780.pdf

Mos, L., Cooper, G. A., Serben, K., Cameron, M. & Koop, B. F. Effects of diesel on survival, growth, and gene expression in rainbow trout (Oncorhynchus mykiss) fry. *Environ Sci Technol* **42**, 2656-2662 (2008). https://doi.org/10.1021/es702215c

Lockhart, W. L., Danell, R. W. & Murray, D. A. J. in *Oil in Freshwater: Chemistry, Biology, Countermeasure Technology* (eds John H. Vandermeulen & Steve E. Hrudey) 335-344 (Pergamon, 1987).

Valero Marketing and Supply Company, (Valero Energy Corporation, San Antonio, Texas, 2014). https://www.valero.com/sites/default/files/2019-12/sds_us_-_102-ghs_diesel_fuels_rev2_5-14_0.pdf.

Tagatz, M. E. Reduced oxygen tolerance and toxicity of petroleum products to juvenile American shad. *Chesapeake Sci.* **2**, 65–71 (1961). https://doi.org/10.2307/1350722

HollyFrontier Refining & Marketing LLC (2014). Accessed July 2025. https://www.allmand.com/content/dam/allmand/na/en_us/files/product-safety-data-sheets/SDS%20No.%20162.pdf.

Rehwoldt, R., Lasko, L., Shaw, C. *et al.* Toxicity study of two oil spill reagents toward Hudson River fish species. *Bull. Environ. Contam. Toxicol.* **11**, 159–162 (1974). https://doi.org/10.1007/BF01684597

Hameed, A.M., Al-Azawi, A. J. M. Toxic Effects Of Acute And Chronic Diesel Fuel On The behavioral And Certain Liver Enzymes In Common Carp (Cyprinus carpio L. 1758). *Iraqi Journal of Science* **Special Issue, Part B**, 263-271 (2016).

Bob-Manuel, F. G. Acute Toxicity Tests of Different Concentrations of Diesel Fuel on the Mudskipper, Periophthalmus koelreuteri (Pallas 1770): (Gobiidae). *Journal of human ecology (Delhi)* **39**, 171-174 (2012). https://doi.org/10.1080/09709274.2012.11906508

Dede, E. B. & Kaglo, H. D. Aqua-toxicological Effects of Water Soluble Fractions (WSF) Of Diesel Fuel On O. Niloticus Fingerlings. *Journal of Applied Sciences and Environmental Management* **5** (2010). https://doi.org/10.4314/jasem.v5i1.54965

Ekanem, A.P., Ekanem, E.A., Efiom, E.E., Nte, S.J. Effects of Diesel on the Behavior and Histopathology of the Gills of Fresh Water Fish (Clarias gariepinus). *International Journal of Engineering and Applied Sciences* **5**, 41-43 (2018).

Juhnke, I. & Lüdemann, D. Results of a study of 200 chemical compounds for acute fish toxicity with the golden orfe test. *Z. Wasser Abwasser Forsch.* **11**, 161–164 (1978). (in German).

Supplemental Table 6. Summary of neuromast counts in control and exposed FHM. Values represent the mean and standard deviation for each treatment group. Control fish exhibited higher neuromast counts compared to diesel-exposed fish.

Group	Mean cranial neuromasts (SD)	Mean tail neuromasts (SD)	Mean Total Body Neuromasts (SD)
Control (n = 8)	489.13 (103.80)	200.88 (49.2)	1157.75 (286.9)
Exposed (n = 8)	285.13 (190.24)	37.8 (45.57)	580.3 (49483.2)

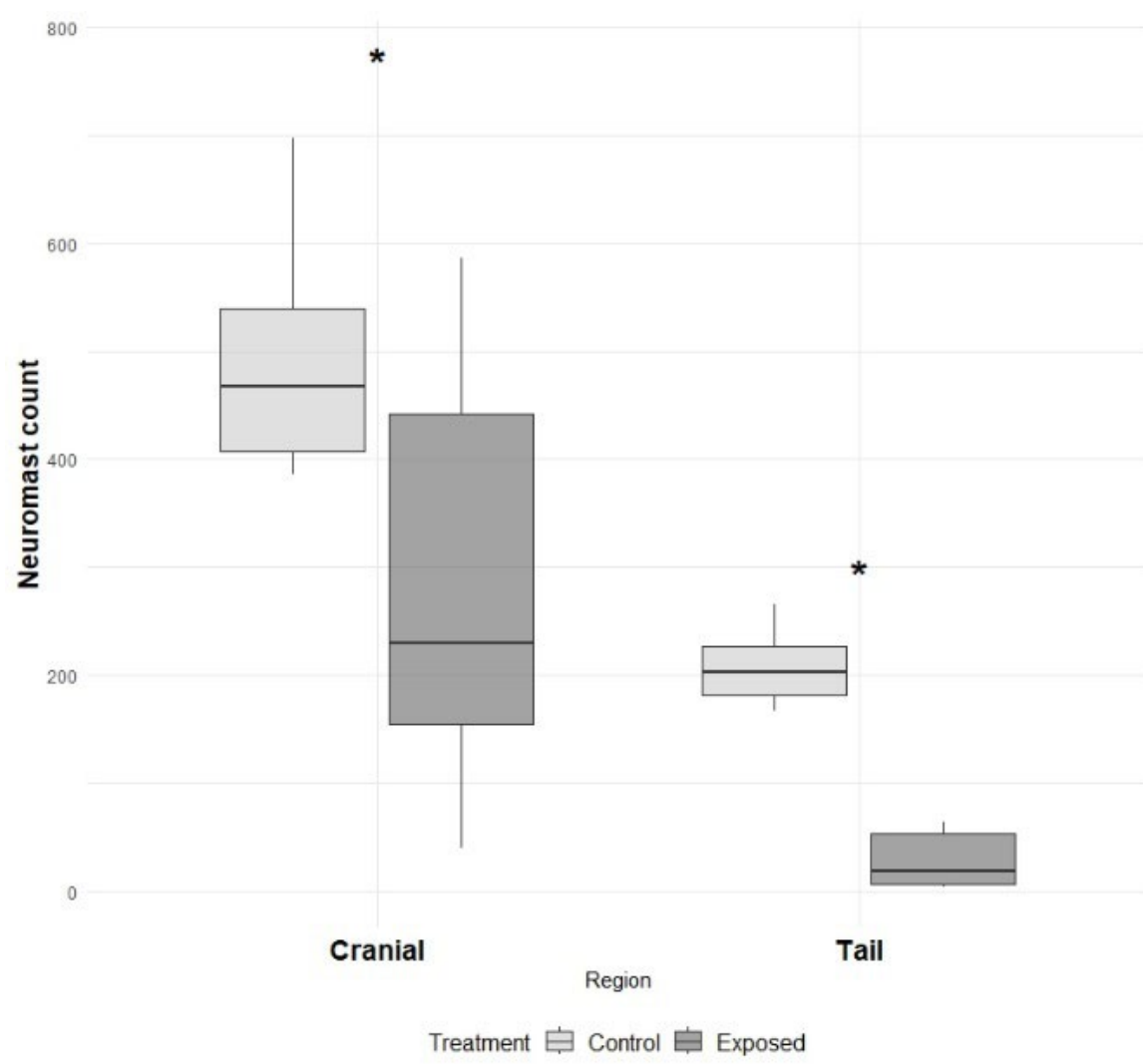

Supplemental Figure 1. Cranial and tail neuromast counts in control vs diesel-exposed Fathead Minnow. Boxplots show median (line), interquartile range (box), and range/SD (whiskers) of neuromast counts. One-tailed Wilcoxon tests (Exposed < Control) revealed significantly fewer neuromasts in exposed fish in the cranial ($p = 0.012$) and tail ($p = 0.046$) regions. Asterisk (*) indicates $p < 0.05$.

Supplemental R code.

```
#Binomial assessment of nonshoaling trials --------------------------------------------
#First we use 2-sided for confidence interval followed by one-sided for p-value

#PMW x GSF nonshoaling
binom.test(19,20)
binom.test(19,20,alternative="greater")
#
#Exact binomial test
#data:  19 and 20
#number of successes = 19, number of trials = 20, p-value = 4.005e-05
#alternative hypothesis: true probability of success is not equal to 0.5
#95 percent confidence interval: 0.7512672 0.9987349
#sample estimates:
#  probability of success                 0.95
#
#Exact binomial test
#data:  19 and 20
#number of successes = 19, number of trials = 20, p-value = 2.003e-05
#alternative hypothesis: true probability of success is greater than 0.5
#95 percent confidence interval: 0.7838938 1.0000000
#sample estimates:
#  probability of success                0.95

#FHM X YEP nonshoaling
binom.test(9,11)
binom.test(9,11,alternative="greater")
#Exact binomial test
#data:  9 and 11
#number of successes = 9, number of trials = 11, p-value = 0.06543
#alternative hypothesis: true probability of success is not equal to 0.5
#95 percent confidence interval: 0.4822441 0.9771688
#sample estimates:
#  probability of success             0.8181818

#Exact binomial test
#data:  9 and 11
#number of successes = 9, number of trials = 11, p-value = 0.03271
#alternative hypothesis: true probability of success is greater than 0.5
#95 percent confidence interval: 0.5299132 1.0000000
```

```
#sample estimates:
#  probability of success            0.8181818

#FHMxCRC nonshoaling
binom.test(8,14)
binom.test(8,14,alternative="greater")

#Exact binomial test
#data:  8 and 14
#number of successes = 8, number of trials = 14, p-value = 0.7905
#alternative hypothesis: true probability of success is not equal to 0.5
#95 percent confidence interval: 0.2886094 0.8233889
#sample estimates:
#  probability of success            0.5714286
#Exact binomial test
#data:  8 and 14
#number of successes = 8, number of trials = 14, p-value = 0.3953
#alternative hypothesis: true probability of success is greater than 0.5
#95 percent confidence interval:  0.3250285 1.0000000
#sample estimates:
#  probability of success            0.5714286

#FHM x GSF nonshoaling
binom.test(14,23)
binom.test(14,23,alternative="greater")

#Exact binomial test
#data:  14 and 23
#number of successes = 14, number of trials = 23, p-value = 0.4049
#alternative hypothesis: true probability of success is not equal to 0.5
#95 percent confidence interval:   0.3854190 0.8029236
#sample estimates:
#  probability of success            0.6086957
#
#Exact binomial test
#data:  14 and 23
#number of successes = 14, number of trials = 23, p-value = 0.2024
#alternative hypothesis: true probability of success is greater than 0.5
#95 percent confidence interval: 0.4168452 1.0000000
#sample estimates:
#  probability of success            0.6086957
```

```
#ALL NONSHOALING TRIALS TOGETHER
binom.test(50,68)
binom.test(50,68,alternative="greater")

#Exact binomial test
#
#data:  50 and 68
#number of successes = 50, number of trials = 68, p-value = 0.0001308
#alternative hypothesis: true probability of success is not equal to 0.5
#95 percent confidence interval: 0.6142896 0.8349615
#sample estimates:
#  probability of success            0.7352941
#Exact binomial test
#data:  50 and 68
#number of successes = 50, number of trials = 68, p-value = 6.542e-05
#alternative hypothesis: true probability of success is greater than 0.5
#95 percent confidence interval: 0.633159 1.000000
#sample estimates:
#  probability of success            0.7352941



# Sign Tests for assessment of shoaling trials ----------------------------------------------------
#In shoaling trials 22 of the 25 predation events were of diesel exposed prey.
#Predator avoidance tanks held 3 exposed and 3 control prey fish or
#4 exposed and 4 control prey fish at the beginning of each trial. We
#use proportion of predation that is of exposed fish for each experimental
#unit in the Sign tests below.

install.packages("BSDA")  # Install the package
library(BSDA)             # Load the package

# All Shoaling Trials including the six FHM x GSF and the two FHC x GSF trials
InData <- data.frame(prop = c(1,1,1,0.66,1,0.66,1,0.66))
SIGN.test(InData$prop, md = 0.5, alternative = "greater")

#One-sample Sign-Test
#
#data:  InData$prop
#s = 8, p-value = 0.003906
```

```
#alternative hypothesis: true median is greater than 0.5
#95 percent confidence interval:
#  0.66  Inf
#sample estimates:
#  median of x
#           1
#
#Achieved and Interpolated Confidence Intervals:
#
#  Conf.Level L.E.pt U.E.pt
#Lower Achieved CI     0.8555   0.66    Inf
#Interpolated CI       0.9500   0.66    Inf
#Upper Achieved CI     0.9648   0.66    Inf


# Shoaling Trials including only the six FHM x GSF trials
InData <- data.frame(prop = c(1,1,1,0.66,1,0.66))
SIGN.test(InData$prop, md = 0.5, alternative = "greater")

#One-sample Sign-Test
#
#data:  InData$prop
#s = 6, p-value = 0.01562
#alternative hypothesis: true median is greater than 0.5
#95 percent confidence interval:
#  0.66  Inf
#sample estimates:
#  median of x
#                  1
#
#Achieved and Interpolated Confidence Intervals:
#
#  Conf.Level L.E.pt U.E.pt
#Lower Achieved CI     0.8906   0.66    Inf
#Interpolated CI       0.9500   0.66    Inf
#Upper Achieved CI     0.9844   0.66    Inf


# Shoaling Trials including only the two FHC x GSF.
# Two trials is not enough to consider a valid analysis in a Sign Test but this
#is how we are presenting our raw data
```

```
InData <- data.frame(prop = c(1,0.66))
SIGN.test(InData$prop, md = 0.5, alternative = "greater")

#One-sample Sign-Test
#
#data:  InData$prop
#s = 2, p-value = 0.25
#alternative hypothesis: true median is greater than 0.5
#75 percent confidence interval:
#  0.66  Inf
#sample estimates:
#  median of x
#          0.83


#Fisher’s Tests of Pathologies--------------------------------------------------------------------------------
--------------------
#Raw Presence Absence Data where C is control tanks and T is diesel exposure tanks
  ~Tank, ~Fish, ~`Gill Acute Necrosis`, ~Keratitis, ~Uveitis,
  "C1","F1","N","N","N",
  "C2","F1","N","N","N",
  "C3","F1","N","N","N",
  "C4","F1","N","N","N",
  "C5","F1","N","N","N",
  "C6","F1","N","N","N",
  "C7","F1","N","N","N",
  "C8","F1","N","N","N",
  "T1","F1","Y","Y","Y",
  "T2","F1","Y","Y","Y",
  "T3","F1","N","Y","Y",
  "T4","F1","Y","Y","Y",
  "T5","F1","N","Y","N",
  "T6","F1","Y","Y","Y",
  "T7","F1","Y","Y","Y",
  "T8","F1","Y","Y","Y"
)
#Fisher’s test for Uveitis
data <- matrix(c(7, 1, 0, 8), nrow = 2, dimnames = list(
  Path = c("Uveitis", "None"),
  Treatment = c("Diesel", "Control")
```

```
))
print(data)
result <- fisher.test(data, alternative = "greater")
print(result)


#Treatment
#Path      Diesel Control
#Uveitis      7       0
#None         1       8
#
#Fisher's Exact Test for Count Data
#data:  data
#p-value = 0.0006993
#alternative hypothesis: true odds ratio is greater than 1
#95 percent confidence interval:
# 4.896026      Inf
#sample estimates:
#odds ratio
#       Inf





# Fisher’s test for Keratitis

data <- matrix(c(8, 0, 0, 8), nrow = 2, dimnames = list(
  Path = c("Keratitis", "None"),
  Treatment = c("Diesel", "Control")
))

print(data)
result <- fisher.test(data, alternative = "greater")
print(result)

# Treatment
# Path        Diesel Control
# Keratitis      8       0
# None           0       8
#
```

```
#Fisher's Exact Test for Count Data
#data:  data
#p-value = 7.77e-05
#alternative hypothesis: true odds ratio is greater than 1
#95 percent confidence interval:
# 9.686976      Inf
#sample estimates:
#odds ratio
#       Inf


# Fisher test for Gill Acute Necrosis (GAN)

data <- matrix(c(8, 0, 2, 6), nrow = 2, dimnames = list(
  Path = c("GAN", "None"),
  Treatment = c("Diesel", "Control")
))

print(data)
result <- fisher.test(data, alternative = "greater")
print(result)

#Treatment
#Path   Diesel Control
#GAN       8       2
#None      0       6
#
#Fisher's Exact Test for Count Data
#data:  data
#p-value = 0.003497
#alternative hypothesis: true odds ratio is greater than 1
#95 percent confidence interval:
# 2.936944      Inf
#sample estimates:
#odds ratio
#       Inf


# Wilcoxon Rang Sum for Neuromast counts ----------------------------------------------
#Neuromast counts on whole body
total_data <- data.frame(
```

```
  Fish_ID = 1:16,
  Group = rep(c("Control", "Exposed"), each = 8),
  Total = c(1651, 1024, 1223, 1380, 955, 761, 940, 1328,
            844, 403, 440, 331, 900, 1522, 142, 60))
wilcox_results <- wilcox.test(Total ~ Group, data = total_data)
print(wilcox_results)
wilcox_results <- wilcox.test(Total ~ Group,
                              data = total_data,
                              alternative = "greater")
print(wilcox_results)

# Wilcoxon rank sum exact test
#
# data:  Total by Group
# W = 55, p-value = 0.007382
# alternative hypothesis: true location shift is greater than 0


#Neuromast counts on Tail
total_data <- data.frame(
  Fish_ID = 1:16,
  Group = rep(c("Control", "Exposed"), each = 8),
  Total = c(266,256,208,198,166,109,187,217,18,4,18,49,64,137,6,7))
wilcox_results <- wilcox.test(Total ~ Group, data = total_data)
print(wilcox_results)
wilcox_results <- wilcox.test(Total ~ Group,
                              data = total_data,
                              alternative = "greater")
print(wilcox_results)

#Wilcoxon rank sum test with continuity correction

#data:  Total by Group
#W = 63, p-value = 0.0006741
#alternative hypothesis: true location shift is greater than 0


#Neuromast counts on Head / cranial region

total_data <- data.frame(
  Fish_ID = 1:16,
```

```
  Group = rep(c("Control", "Exposed"), each = 8),
  Total = c(698,547,456,538,410,400,386,478,470,163,261,199,432,586,130,40))
wilcox_results <- wilcox.test(Total ~ Group, data = total_data)
print(wilcox_results)
wilcox_results <- wilcox.test(Total ~ Group,
                              data = total_data,
                              alternative = "greater")
print(wilcox_results)

#Wilcoxon rank sum exact test
#
#data:  Total by Group
#W = 50, p-value = 0.03248
#alternative hypothesis: true location shift is greater than 0


# Wilcoxon Rank Sum Test for Interlamellar Filling Of Gills ----------------------------------------
----

#Interlamellar Filling of Gills
#Suggests no difference

total_data <- data.frame(
  Fish_ID = 1:16,
  Group = rep(c("Control", "Exposed"), each = 8),
  Total = c(2,3,1,1,1,2,2,1,1,2,1,1,1,1,2,2))
wilcox_results <- wilcox.test(Total ~ Group, data = total_data)
print(wilcox_results)

#Wilcoxon rank sum test with continuity correction
#
#data:  Total by Group
#W = 37.5, p-value = 0.5501
#alternative hypothesis: true location shift is not equal to 0
```
